# KIF23 is a potential biomarker of diffuse large B cell lymphoma: Analysis based on bioinformatics and immunohistochemistry

**DOI:** 10.1097/MD.0000000000029312

**Published:** 2022-06-17

**Authors:** Yuqi Gong, Lingna Zhou, Liya Ding, Jing Zhao, Zhe Wang, Guoping Ren, Jing Zhang, Zhengrong Mao, Ren Zhou

**Affiliations:** a Department of Pathology, The First Affiliated Hospital, Zhejiang University School of Medicine, Hangzhou, China; b Department of Pathology and Pathophysiology, Institute of Pathology and Forensic Medicine, Zhejiang University School of Medicine, Hangzhou, China; c Department of Pathology, Sir Run Run Shaw Hospital, Zhejiang University School of Medicine, Hangzhou, China; d Department of Pathology, Xijing Hospital, Fourth Military Medical University, Xi’an, China.

**Keywords:** biomarker, DLBCL, KIF23, methylation, prognostic value

## Abstract

Diffuse Large B Cell Lymphoma (DLBCL), the most common form of blood cancer. The genetic and clinical heterogeneity of DLBCL poses a major barrier to diagnosis and treatment. Hence, we aim to identify potential biomarkers for DLBCL.

Differentially expressed genes were screened between DLBCL and the corresponding normal tissues. Kyoto Encyclopedia of Genes and Genomes and Gene oncology analyses were performed to obtain an insight into these differentially expressed genes. PPI network was constructed to identify hub genes. survival analysis was applied to evaluate the prognostic value of those hub genes. DNA methylation analysis was implemented to explore the epigenetic dysregulation of genes in DLBCL.

In this study, Kinesin family member 23 (KIF23) showed higher expression in DLBCL and was identified as a risk factor in DLBCL. The immunohistochemistry experiment further confirmed this finding. Subsequently, the univariate and multivariate analysis indicated that KIF23 might be an independent adverse factor in DLBCL. Upregulation of KIF23 might be a risk factor for the overall survival of patients who received an R-CHOP regimen, in late-stage, whatever with or without extranodal sites. Higher expression of KIF23 also significantly reduced 3, 5, 10-year overall survival. Furthermore, functional enrichment analyses (Kyoto Encyclopedia of Genes and Genomes, Gene oncology, and Gene Set Enrichment Analysis) showed that KIF23 was mainly involved in cell cycle, nuclear division, PI3K/AKT/mTOR, TGF-beta, and Wnt/beta-catenin pathway in DLBCL. Finally, results of DNA methylation analysis indicated that hypomethylation in KIF23's promoter region might be the result of its higher expression in DLBCL.

The findings of this study suggested that KIF23 is a potential biomarker for the diagnosis and prognosis of DLBCL. However, further studies were needed to validate these findings.

## 1 Introduction

Diffuse Large B-cell Lymphoma (DLBCL) is the most common type of hematological cancer, accounting for 30%–40% of non-Hodgkin lymphoma.^[1]^ The standard treatment is chemoimmunotherapy with rituximab plus cyclophosphamide, doxorubicin, vincristine, and prednisone (R-CHOP), leading to the cure or remission of 60% of patients. However, 40% of patients succumb to DLBCL.^[2]^ The application of next-generation sequencing has revealed a great degree of molecular and clinical heterogeneity in DLBCL.^[3]^ This heterogeneity poses a series of challenges in the understanding and treatment of DLBCL. Further deciphering genes and signaling pathways involved in the initiation and development of DLBCL may provide a chance for efficient therapy.

Kinesin superfamily proteins possess a highly conserved motor domain, which hydrolyzes ATP to generate energy leading to a conformational change in their movement.^[4]^ Those proteins participate in multiple biological functions, including mitosis, organelles transport, and signaling events.^[5,6]^ Dysregulation of Kinesin superfamily proteins is involved in the initiation, development, and progress of human cancers.^[7]^ Kinesin family member 23 (KIF23), the member of kinesin 6 family, located at the interzone of mitotic spindles, plays a critical role in cytokinesis.^[7,8]^ Tumor suppressor gene p53 can repress KIF23 transcription by downregulation of KIF23 promoter activity,^[9]^ while TCF-4 can directly bind to the promoter of KIF23 at -814/-805 bp (GGGTCAAAGA) to activate its transcription.^[10]^ KIF23 knockdown significantly decreased the proliferation of glioma cells^[10]^ and gastric cancer cells^[11]^ in vitro and in vivo. In Synovial Sarcoma, KIF23 was involved in metastasis, leading to reduced survival.^[12]^ A recent study found that a lncRNA PVT1 knockdown reduced KIF23 expression by enhancing miR-15a-5p, thereby attenuating prostate cancer progression.^[13]^ Nonetheless, the roles of KIF23 in DLBCL remain unclear.

In this study, we selected four microarray datasets to screen differentially expressed genes (DEGs) between DLBCL and the corresponding normal tissues. Kyoto Encyclopedia of Genes and Genomes (KEGG) and Gene oncology (GO) analyses were performed to obtain insights into these DEGs. Protein protein interaction (PPI) network was constructed to identify hub genes. Next, survival analyses identified that KIF23 was significantly associated with poor prognosis in DLBCL based on four datasets. Finally, LinkedOmics, KEGG, GO, Gene Set Enrichment Analysis (GSEA), and methylation array of TCGA dataset were all used to obtain the possible molecular mechanisms of KIF23. Our results improve the understanding of the roles and mechanisms of KIF23 in DLBCL.

## 2 Materials and methods

### 2.1 Data source

To obtain key genes related to DLBCL, we used “DLBCL” and “RNA” as keywords to search gene expression profiles in GEO database. Datasets containing normal tissues and DLBCL samples were our choice. Finally, four GEO datasets (GSE25638, GSE44337, GSE56315, GSE32018) were selected for further study. GSE25638, GSE44337 and GSE56315 were based on GPL570 ([HG-U133_Plus_2] Affymetrix Human Genome U133 Plus 2.0 Array). GSE32018 was based on GPL6480 (Agilent-014850 Whole Human Genome Microarray 4 × 44 K G4112F (Probe Name version)). The data for GSE25638, GSE44337, GSE56315 and GSE32018 consisted of 26 DLBCL patients vs 13 controls, 9 DLBCL patients vs 3 controls, 55 DLBCL patients vs 33 controls and 22 DLBCL patients vs 7 controls respectively. Two DNA methylation profiles (TCGA, n = 48; GSE92679, n = 97) were selected for methylation analysis.

Three GEO datasets (GSE10846,^[14]^ GSE32918,^[15]^ GSE23501^[16]^) were selected for survival analysis. GSE10846, GSE32918, and GSE23501 consist of 414 DLBCL patients (181 patients received CHOP regimen and 233 patients received RCHOP regimen), 244 DLBCL patients, and 69 DLBCL patients, respectively. For multivariate cox analysis, several clinical factors (age, gender, regimen, ECOG, stage, LDH ratio, and extranodal sites) were included.

27 DLBCLs paraffin-embedded tissues and 18 lymphoid samples were obtained from Second hospital of Shaoxing and the First Affiliated Hospital of Zhejiang University, respectively, with the necessary informed consent of patients (samples were collected from 2015–2016). Another 77 DLBCLs paraffin-embedded tissues with clinical information were collected from the First Affiliated Hospital of Zhejiang University (samples were collected from 2009–2016).

### 2.2 DEG identification

DEG identification was performed as follows. First, we mapped the probe IDs to the gene symbols using R software (version 4.0.0). Then, the limma package (version 3.44.3) was adopted to identify DEGs between DLBCL and the corresponding normal controls. Genes with false discovery rate (FDR) adjusted *P* < .05 and |log2 Fold change| > 1 were considered as DEGs. Finally, the overlapping DEGs, shared by all the four datasets (GSE25638, GSE44337, GSE56315, GSE32018), were obtained and visualized using VennDiagram (version 1.6.20).

### 2.3 KEGG pathway and GO annotation analysis

To reveal the potential functions of overlapping DEGs, we performed enrichment analyses as follows. First, gene symbols were mapped to ENTREZID using the R package org.Hs.eg.db (version 3.11.4). Then, we conducted KEGG pathway and GO annotation analyses by using the R package clusterProfiler (version, 3.16.1) with the “enrichKEGG” and “enrichGO” functions, respectively. GO terms consist of biological process, cellular component, and molecular function. False discovery rate (FDR) adjusted *P* < .05 was regarded statistically significant. Finally, the function “Dotplot” was used to visualize.

### 2.4 PPI network and hub genes

Search Tools for Retrieval of Interacting Genes (STRING) provides a platform for constructing protein association networks. Overlapping DEGs were submitted to STRING. The combined score of PPI pairs was no less than .4. We used the plugin cytoHubba of Cytoscape (3.8.0) to calculate and visualize hub genes in the PPI network. The Local-based method, including four algorithms: Degree method (Geg), Maximal Clique Centrality, Density of Maximum Neighborhood Component, and Maximum Neighborhood Component, of cytoHubba, was used to screen out top 30 hub genes.

VennDiagram (1.6.20) was then applied to identify overlapping hub genes calculated by these four algorithms. We used Gene Expression Profiling Interactive Analysis (GEPIA, http://gepia.cancer-pku.cn/)^[17]^ to validate the differential expression levels of these overlapping hub genes.

### 2.5 Survival analysis for hub genes

We performed Kaplan-Meier analyses to explore associations between overall survival (OS) and hub genes. GSE10846 and GSE32918 were used as test cohorts. GSE23501 was used as the validation cohort. For further study, as GSE10846 provided detailed and refined information about each patient, Kaplan-Meier, univariate analyses, and multivariate analysis, based on GSE10846, were used to explore the relationship between KIF23 and prognosis patients under different clinical conditions. Patients with missing value or loss of follow-up were excluded. Two R packages (survival,version 3.1-12; survminer, version 0.4.7) were used for Kaplan-Meier, univariate analyses, and multivariate analysis.

### 2.6 Immunohistochemistry analysis

IHC staining was performed with the antibody KIF23 (Abcam, ab235955, 1:550) following the manufacturer's protocol. The staining value of KIF23 was calculated as previously described^[18,19]^: the staining index (values 0–12), obtained as the intensity of KIF23-positive staining (3, strong staining; 2, moderate staining; 1, weak staining; 0, no staining) and the proportion of immuno-positive cells of interest (4, >75%; 3, 51%–75%; 2, 26%–50%; 1, <25%), was calculated. For example, if a patient specimen showed strong KIF23-positive staining in more than 75% cells, the staining score was 12 (3 multiplied by 4 equals 12). For survival analysis, all patients were divided into two groups according to the median value of the KIF23 staining index: KIF23-high expression group (≥median) and KIF23-low expression group (<median).

### 2.7 LinkedOmics and GSEA

LinkedOmics (http://www.linkedomics.org/login.php) provides a platform for analyzing multi-dimensional datasets of TCGA.^[20]^ We applied Pearson's correlation coefficient to detect KIF23 co-expression genes. KEGG pathway and GO (Biological Process) were performed to obtain deeper insights into the potential functions of KIF23 in DLBCL. In addition, All the 48 DLBCL patients from TCGA were separated into two groups according to the KIF23 median value. GSEA (4.0.3)^[21]^ was then performed. In this study, we chose h.all.v7.2.symbols.gmt [Hallmarks] as the gene set database. The number of permutations was 1000, and the permutation type was phenotype.

### 2.8 The combined analysis of RNA-seq and corresponding DNA methylation microarray of DLBCL from TCGA

To explore the possible reason for the higher expression of KIF23 in DLBCL, we combined RNA-seq and the corresponding DNA methylation profile for analysis. Both datasets from TCGA were downloaded from UCSC Xena. DNA methylation profile GSE92679 was selected to validate the hypomethylation in the promoter region of KIF23. We used Pearson correlation and Spearman correlation analysis to determine the relationship between methylation sites of the KIF23 promotor region and KIF23 mRNA expression. *P* < .05 and *r* > 0.3 were regarded as the standard of the significant correlation.

### 2.9 Statistical analysis

The student's t-test was applied to evaluate the difference in KIF23 expression between DLBCL tissues and lymphoid tissues. The association between KIF23 expression and clinical factors was analyzed by chi-square test. Kaplan-Meier analysis was used to compare the OS between KIF23-high and KIF23-low groups. Univariate cox analysis was applied to calculate the relationship of OS with clinical factors and KIF23 expression in DLBCL. Multivariate cox analysis was performed to confirm the prognostic value of KIF23 in DLBCL by including all the parameters with *P* < .05 in univariate cox analysis. R (version 4.0.0) was used to perform statistical analysis. The *P* < .05 was considered statistically significant.

## 3 Results and discussion

### 3.1 DEG identification

**Figure 1:**
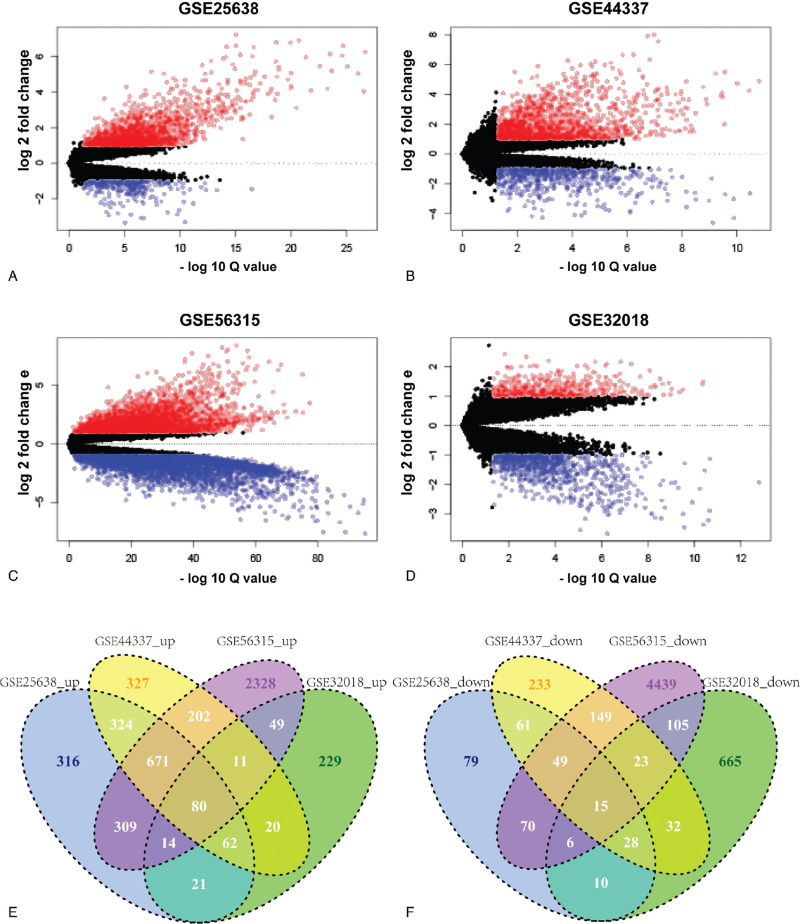
Identification of DEGs in DLBCL. Volcano plot of DEGs between DLBCL and noncancerous tissues in GSE25638 (A), GSE44337 (B), GSE56315 (C), GSE32018 (D). The red points represent significantly upregulated genes and the green points represent significantly downregulated genes. (E) Overlapping genes of significantly upregulated genes among the 4 datasets mentioned above. (F) Overlapping genes of significantly downregulated genes among the 4 datasets mentioned above.

Four datasets were used to identify DEGs between DLBCL and the corresponding normal tissues. For GSE25638, 1787 upregulated genes and 318 downregulated genes were obtained (Fig. 1A). For GSE44337, 1697 upregulated genes and 590 downregulated genes were obtained (Fig. 1B). For GSE56315, 3665 upregulated genes and 4856 downregulated genes were obtained (Fig. 1C). For GSE32018, 486 upregulated genes and 884 downregulated genes were obtained (Fig. 1D). Finally, 80 overlapping genes were significantly upregulated (Fig. 1E), and 15 overlapping genes were remarkably downregulated (Fig. 1F) in DLBCL compared to normal tissues (Table 1). The log fold change and the *P* value of these overlapping genes were listed in supplementary file 1.

**Table 1:** The common DEGs of four gene expression profiles (*P*-value < .05, |logFC| >1.0).

Common DEGs	Gene symbol
Upregulated DEGs	LUM, GPNMB, CHI3L1, VNN1, CEP55, FNDC3B, CXCL11, CTSD, PPA1, DTL, C17orf58, DEPDC1B, DSCC1, KIF4A, TRIP13, POSTN, LGALS3, ECT2, KIF23, AIMP2, BRCA1, LDHA, KIF15, MELK, TOP2A, MAD2L1, LRRC59, TYMS, DLGAP5, NCAPH, FAM83D, ANLN, BUB1, BYSL, PBK, LRR1, OIP5, BCAT1, KIF14, SPC24, KIF2 C, TTK, MRPL15, CDK1, IGF2BP3, CDC20, ASF1B, CCNB1, ZWINT, RAD51AP1, PCNA, CDCA8, GINS1, NEK2, ASPM, GMNN, BUB1B, SHCBP1, CCNE2, HMMR, CDT1, NUF2, TFRC, RAD51, AHCY, SGTB, STIL, CENPF, PGD, POLQ, ZNF185, DEPDC1, ATF3, MYBL2, MCM6, CDCA7, RGS13, FRK, CYB5R2, ADA
Downregulated DEGs	IGHD, RIC3, JUN, GNG7, OTUD1, CCR6, RASGRP2, GRAP, C12orf42, FCRL1, DPEP2, GAPT, GLIPR1, CYSLTR1, FCER2

DEGs = differentially expressed genes.

### 3.2 KEGG pathway and GO annotation enrichment analyses

**Figure 2:**
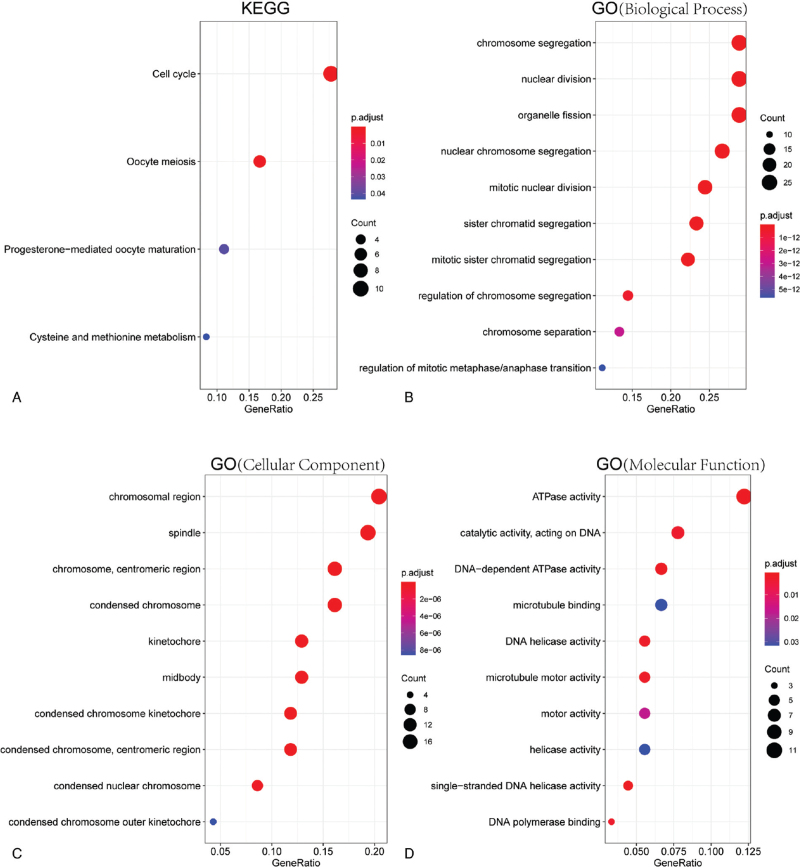
Function enrichment analysis of DEGs in DLBCL. The KEGG pathways (A), GO (Biological Process) (B), GO (Cellular Component) (C), and GO (Molecular Function) (D) in the enrichment analysis of 95 DEGs in DLBCL.

Next, Two R packages (clusterProfiler, org.Hs.eg.db) were applied to conduct KEGG pathway and GO annotation enrichment analyses of these 95 overlapping DEGs. The top four KEGG enrichment pathways were cell cycle, oocyte meiosis, progesterone-mediated oocyte maturation, and cysteine and methionine metabolism (Fig. 2A). The GO biological process analysis revealed that these 95 common DEGs were significantly enriched in chromosome segregation, nuclear division, organelle fission (Fig. 2B). The GO cellular component analysis showed that chromosomal region, spindle, centromeric region kinetochore were markedly enriched (Fig. 2C). Besides, the top three GO molecular function terms were ATPase activity, catalytic activity acting on DNA, DNA-dependent ATPase activity (Fig. 2D).

### 3.3 PPI network and hub genes identification

**Figure 3:**
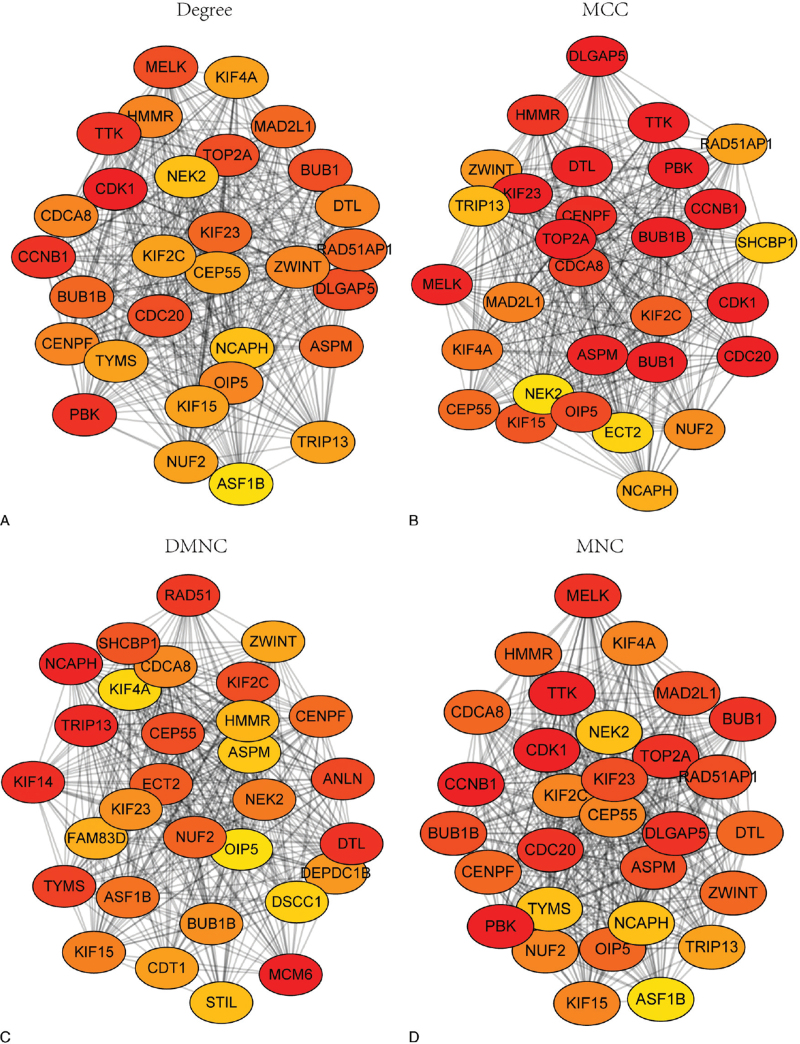
PPI network of hub genes. The top 30 hub genes identified by Degree method (Geg) (A), Maximal Clique Centrality (MCC) (B), Density of Maximum Neighborhood Component (DMNC) (C), and Maximum Neighborhood Component (MNC) (D) respectively. The deeper the color, the higher the gene rank.

**Figure 4:**
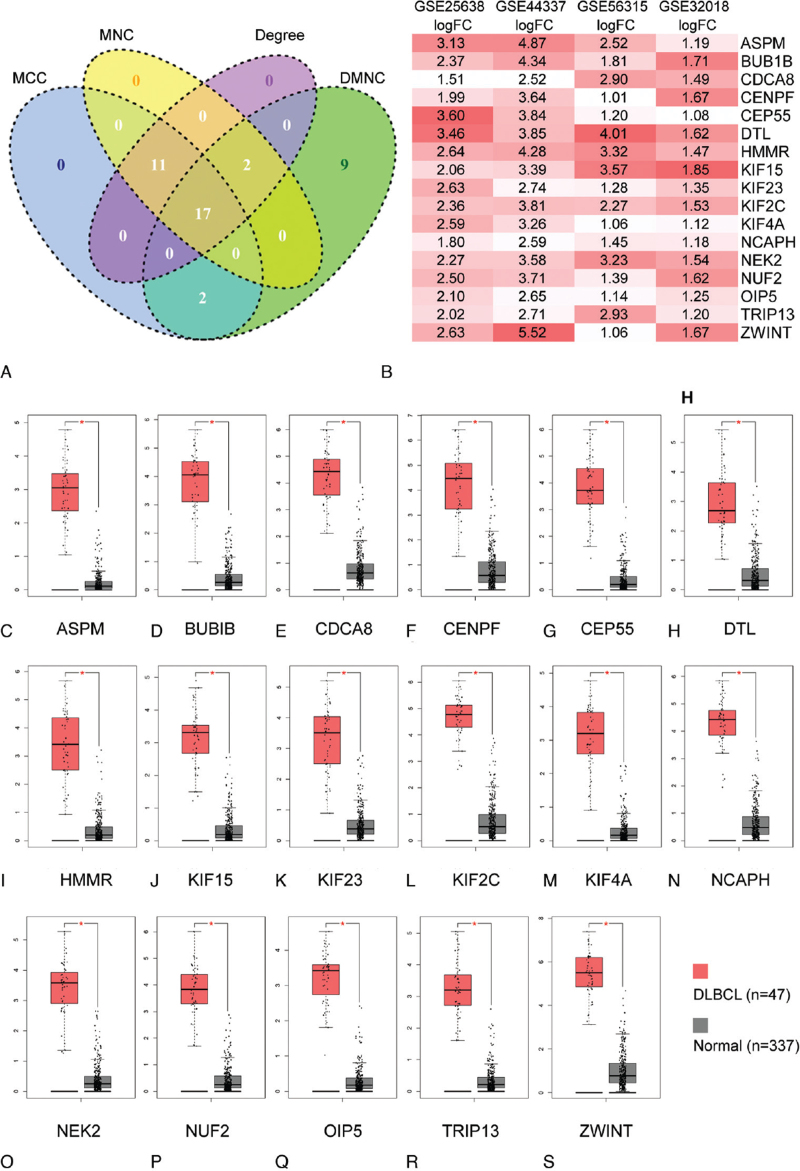
Identifications of the overlapping hub genes. (A) The overlapping hub genes of four algorithms. (B) Foldchange of the overlapping hub genes in four datasets. (C) Expression levels of the overlapping hub genes between DLBCL and normal tissues in GEPIA database.

STRING database was used to construct the protein-protein interaction network among the 95 common DEGs. A plugin cytoHubba of Cytoscape (3.8.0) was applied to identify and visualize the top 30 hub genes. As the heterogeneity of protein network, it is reasonable to use more than one algorithm to catch hub genes. Since the local-based method (including four algorithms: Degree method (Geg), Maximal Clique Centrality, Density of Maximum Neighborhood Component, and Maximum Neighborhood Component) of cytoHubba considers the relationship between the node and its direct neighbors, we used all the four algorithms of this method to identify the top 30 hub genes (Fig. 3A-D). Then, 17 overlapping hub genes were obtained (Fig. 4A). All the 17 hub genes were upregulated in DLBCL compared to normal tissues in the 4 GEO datasets (Fig. 4B). Similar results were obtained from the GEPIA database (Fig. 4C-S).

### 3.4 Relationship between hub genes and prognosis of DLBCL patients

**Figure 5:**
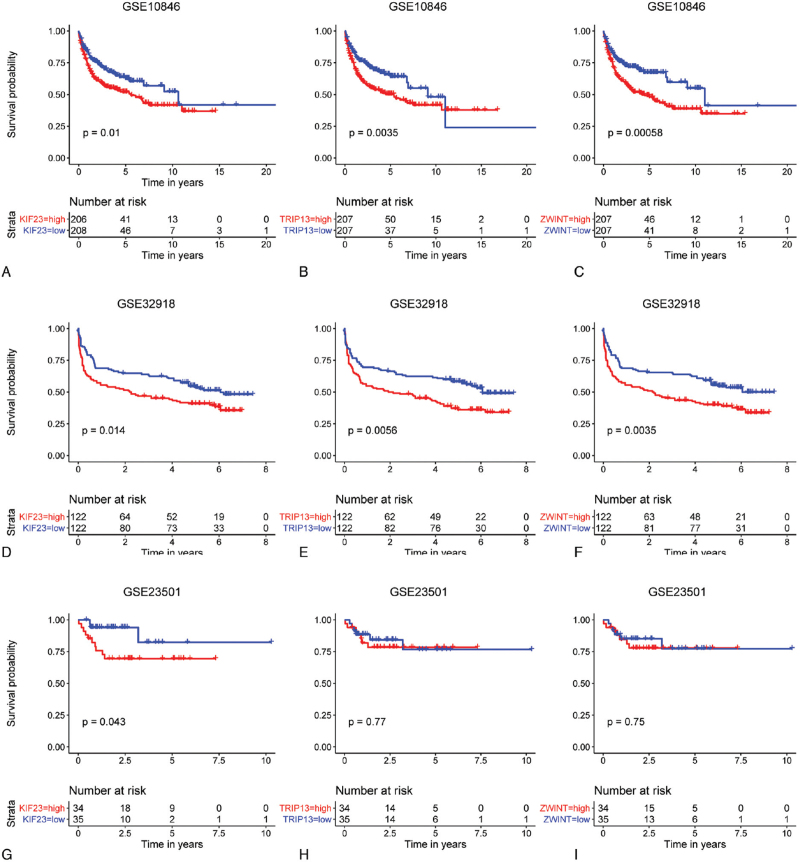
Overall survival of DLBCL patients. The Kaplan-Meier curve was performed according to the median value of the gene expression level (high: median value or higher; low: less than median value) in GSE10846 (A-C), GSE32918 (D-F), and GSE23501 (G-I). *P* < .05 showed statistical significance.

For OS, two datasets, GSE10846 consisted of 414 DLBCL cases, GSE32918 consisted of 244 DLBCL cases, was used for Kaplan-Meier analysis. Among these 17 hub genes, three genes, KIF23 (*P* = .01, *P* = .01), TRIP13 (*P* < .01, *P* < .01), and ZWINT (*P* < .001, *P* < .01), were significantly associated with shorter lives in both datasets (Fig. 5A-F). Next, GSE23501(n = 69), were used to validate the results. In this dataset, patients with higher expression of KIF23 had poor prognosis (*P* = .04), while patients with different expression levels of TRIP13 (*P* = .77) or ZWINT (*P* = .75) showed no significant differences in prognosis (Fig. 5 G-I). Therefore, KIF23 is considered as a critical gene in DLBCL.

### 3.5 IHC validation of KIF23 importance in DLBCL

**Figure 6:**
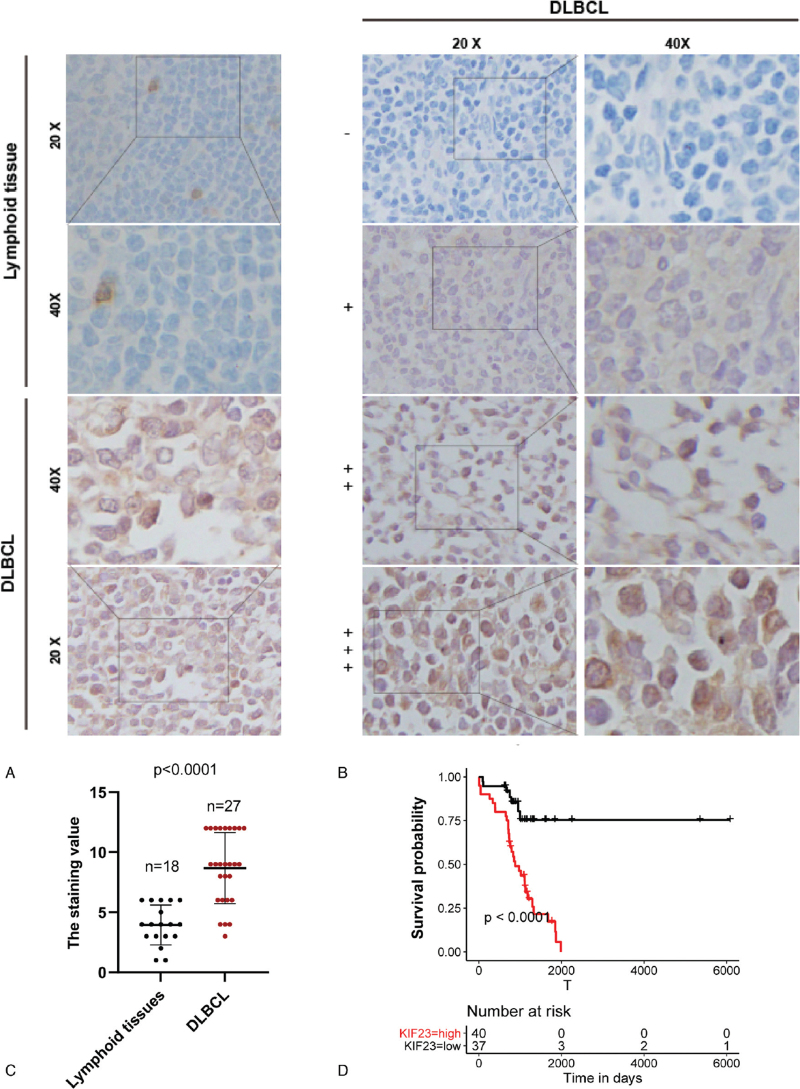
Overexpression of KIF23 and its prognostic value in DLBCL. (A) Staining images of KIF23 protein expression in lymphoid tissue and DLBCL sample. (B) Statistic of KIF23 protein expression in lymphoid tissue and DLBCL sample. (C) Staining images of KIF23 protein expression in DLBCL patients (negative: -, weak: +  moderate: ++, strong: ++ + ). (D) The Kaplan-Meier curve was performed between KIF23 higher group (++/ + + + ) and lower group (−/ + ) of 77 DLBCL patients.

We then identified the KIF23 expression level in 45 samples, including 17 lymph nodes and 27 DLBCL samples. IHC experiment validated that DLBCL samples showed higher expression of KIF23 compared to lymphoid tissues (Fig. 6A-C). We classified DLBCL patients into four categories (Fig. 6B, the staining value ≥ 9: +++; the staining value≥4: ++; the staining value≥1: +  the staining value = 0: −). Furthermore, 77 DLBCL patients with clinical information were used for survival analysis. We separate all patients into two groups according to the median value of the KIF23 staining index: KIF23-high expression group (≥median value) and KIF23-low expression group (<median value). The clinical information and KIF23 staining value of those 77 DLBCL paraffin-embedded tissues were detailed in supplementary file 2. Kaplan-Meier analysis suggested that higher expression of KIF23 was significantly associated with poor prognosis in DLBCL (Fig. 6D).

### 3.6 KIF23 as an independent prognostic factor in DLBCL

**Figure 7:**
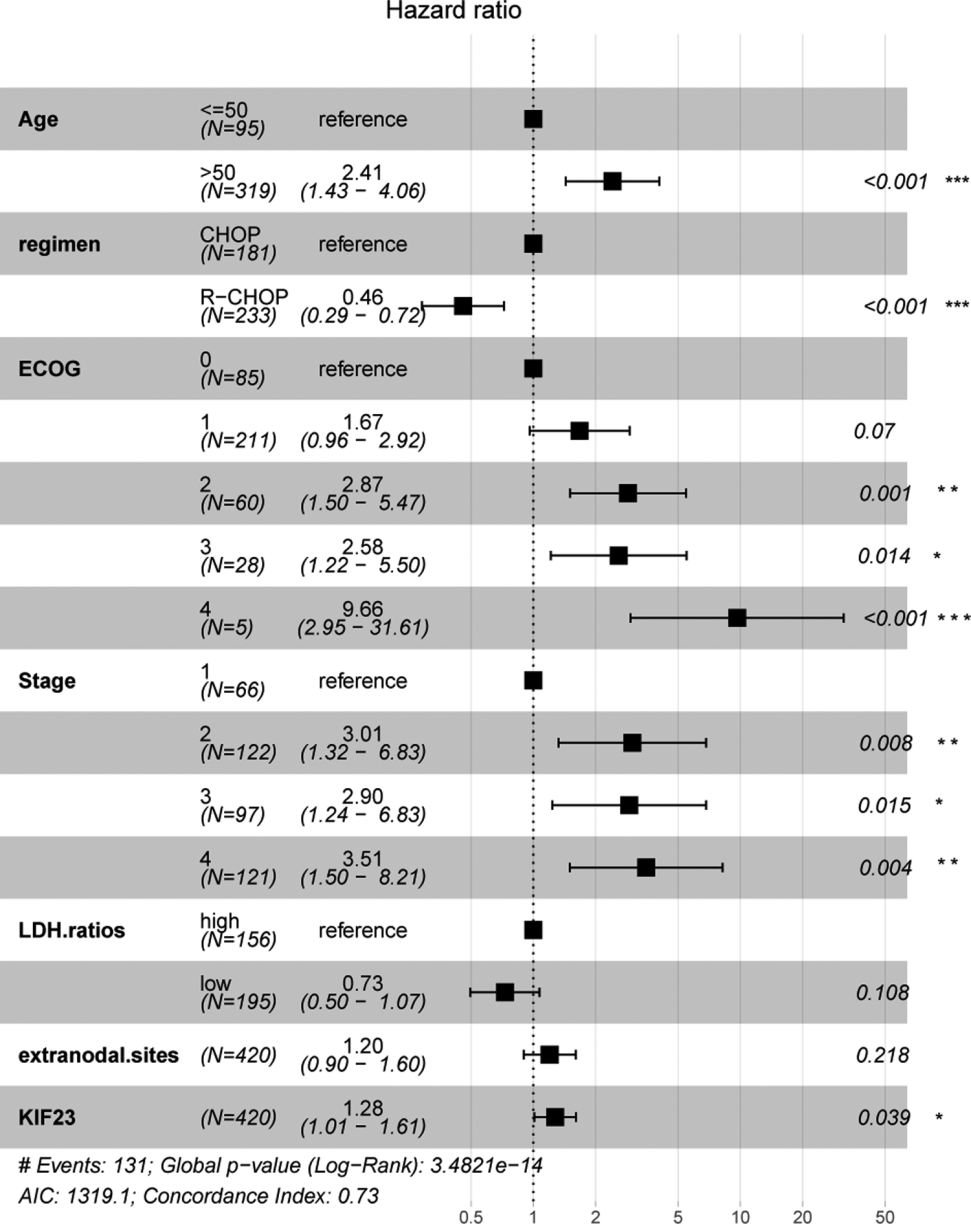
Multivariate cox analysis of significant clinical factors in DLBCL.

To identify the importance of KIF23 in DLBCL, we used information from GSE10846 for further studies because this dataset had detailed information on clinical and treatment attributes. Univariate analysis indicated that age (HR 1.03; CI 1.018–1.041; *P* < .001;), regimen (HR 0.53; CI 0.376–0.719; *P* < .001), ECOG (HR 1.82; CI 1.551–2.136; *P* < .001), stage (HR 1.51; CI 1.293–1.758; *P* < .001), LDH ratio (HR 1.14; CI 1.095–1.181; *P* < .001), extranodal sites (HR 1.21; CI 1.001–1.452; *P* < .001), KIF23 (HR 1.36; CI 1.101–1.690; *P* < .001) significantly correlated with OS (Table 2). However, males and females showed no significantly different outcomes in DLBCL (HR 1.02; CI 0.744–1.402; *P* = .89). Follow-up evaluation of multivariate analysis of these significantly clinical factors demonstrated that older age (HR 2.41; CI 1.43–4.06; *P* < .001), ECOG2 (HR 2.87; CI 1.50–5.47; *P* < .01), ECOG3 (HR 2.58; CI 1.22–5.50; *P* = .01), ECOG4 (HR 9.66; CI 2.95–31.61; *P* < .001), stage 2 (HR 3.10; CI 1.32–6.83; *P* < .01), stage 3 (HR 2.90; CI 1.24–6.83; *P* = .02), stage 4 (HR 3.51; CI 1.50–8.21, *P* < .01), KIF23 (HR 1.28 CI 1.01–1.61; *P* = .04) were independent risk factors for poor prognosis. Treatment of R-CHOP regimen (HR 0.45; CI 0.28–0.71; *P* < .001) can prolong the survival time of patients (Fig. 7). Results from univariate and multivariate analysis indicated that KIF23 can be an independent risk factor for poor prognosis in DLBCL.

**Table 2:** Univariate Cox regression proportional hazards model to analyse KIF23 expression and clinical parameters in DLBCL.

	Univariate analysis		
Variables	HR	95%CI of HR	*P*
Gender (male vs female)	1.02	0.744–1.402	8.97e-01
Age	1.03	1.018–1.041	**3.67e-07**
Regimen (R-CHOP vs CHOP)	0.52	0.376–0.719	**7.71e-05**
ECOG	1.82	1.551–2.136	**2.23e-13**
Stage	1.51	1.293–1.758	**1.56e-07**
LDH ratio	1.14	1.095–1.181	**2.82e-11**
Extranodal sites	1.21	1.001–1.452	**4.85e-02**
KIF23	1.36	1.101–1.690	**4.54e-03**

DLBCL = diffuse large B cell lymphoma, KIF23 = kinesin family member 23.

### 3.7 Relationship between KIF23 and prognosis of patients under different clinical conditions

**Figure 8:**
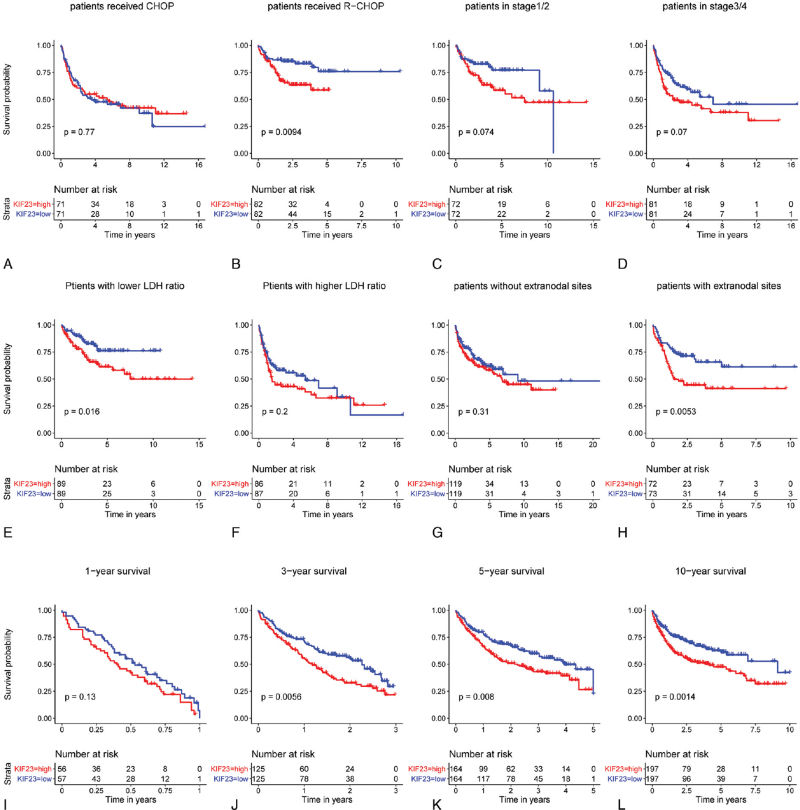
Overall survival of DLBCL patients with different KIF23 expression under different clinical conditions. The Kaplan-Meier curve was performed between KIF23 higher group and lower group of patients who received CHOP regimen (A) or R-CHOP regimen (B), in early-stage (C) or late-stage (D), with lower LDH ratio (E) or higher LDH ratio (F), without extranodal sites (G) or with extranodal sites (H). 1-year (I), 3-year (J), 5-year (K), and 10-year (L) survival between patients in KIF23 higher group and KIF23 lower group.

Then, we explored the effect of KIF23 in patients under different clinical conditions. Kaplan-Meier analysis revealed that the higher expression of KIF23 was associated with inferior prognosis in patients who received the R-CHOP regimen (*P* < .01 Fig. 8B). The HR for R-CHOP was 1.56 (CI 1.046–2.314, *P* = .03, Table 3), suggesting that KIF23 may be a negative prognostic indicator for patients who received R-CHOP regimen, while whatever Kaplan-Meier (*P* = .77) or univariate analysis (HR 1.23, CI 0.87–1.742, *P* = .24), KIF23 expression level showed no significant effect on prognosis in patients received CHOP regimen (Fig. 8A, Table 3). In the early-stage (stage 1 and stage 2) and late-stage (stage 3 and stage 4), Kaplan-Meier analysis indicated that there were no differences in prognosis between KIF23 higher group and KIF23 lower group (*P* = .07, *P* = .07 respectively, Fig. 8C-D). Univariate analysis indicated similar results (HR 1.40, CI 0.8918–2.188, *P* = .14, Table 3) in the early-stage. However, the HR for late-stage was 1.41 (CI 1.057–1.878, *P* = .02 Table 3) indicated that the higher expression of KIF23 might be a prognostic risk factor in patients of late stage. Higher expression of KIF23 was significantly associated with poor clinical outcomes (*P* = .02, Fig. 8E) in patients with lower LDH ratio, whereas there was no notable difference in patients with higher LDH ratio (*P* = .2, Fig. 8F). Univariate analysis suggested no apparent differences between KIF23 higher expression and KIF23 lower expression in patients with lower LDH ratio or higher LDH ratio (HR 1.53 CI 0.9983–2.339, *P* = .51, HR 1.20 CI 0.9219–1.552, *P* = .18 respectively, Table 3). Patients with KIF23 higher expression showed shorter survival time in the group with extranodal sites (*P* < .01, Fig. 8H). Univariate analysis showed similar results (HR 1.40 CI 1.002–1.966, *P* = .04, Table 3). In groups without extranodal sites, the result from Kaplan-Meier analysis showed no significant difference in prognosis between KIF23 higher expression and lower expression (*P* = .31, Fig. 8G). On the contrary, univariate analysis revealed that HR was 1.37 (CI 1.023–1.842, *P* = .03, Table 3), indicating that the higher expression of KIF23 indicated poor prognosis in patients without extranodal sites. Moreover, a subgroup analysis revealed that upregulation of KIF23 was a prognostic risk factor for reduced 3 years (*P* < .01, Fig. 8J; HR 1.10, CI 0.799–1.513, *P* = .56, Table 4), 5 years (*P* < .01, Fig. 8K; HR 1.28, CI 1.027–1.589, *P* = .03, Table 4) and 10 years (*P* < .01, Fig. 8L; HR 1.41, CI 1.141–1.734, *P* < .01, Table 4), but not 1 year (*P* = .13, Fig. 8I; HR 1.45, CI 1.170–1.799, *P* < .001, Table 4) OS in DLBCL patients.

**Table 3:** Correlation between KIF23 and prognosis of patients with different clinical condition.

	OS (n = 420)		
Items	HR	95%CI of HR	*P*
Regimen			
CHOP (n = 142)	1.23	0.87–1.742	.241
RCHOP (n = 164)	1.56	1.046–2.314	**.0293**
Stage			
I/II (n = 144)	1.40	0.8918–2.188	.144
III/IV(n = 162)	1.41	1.057–1.878	**.0193**
LDH ratio			
Low (n = 178)	1.53	0.9983–2.339	.0509
High (n = 173)	1.20	0.9219–1.552	.178
Extranodal sites			
No (n = 238)	1.37	1.023–1.842	**.0345**
Yes (n = 145)	1.40	1.002–1.966	**.0489**

KIF23 = Kinesin family member 23.

**Table 4.** Univariate analysis of correlation between KIF23 and prognosis of patients grouped by 1-year, 3-year, 5-year and 10-year survival in DLBCL.

	Univariate analysis		
Variables	HR	95%CI of HR	*P*
1-year survival	1.10	0.799–1.513	.559
3-year survival	1.28	1.027–1.589	**.0276**
5-year survival	1.41	1.141–1.734	**.00139**
10-year survival	1.45	1.170–1.799	**.000696**

DLBCL = diffuse large B cell lymphoma, KIF23 = kinesin family member 23.

### 3.8 Relationship between KIF23 and clinical features

In addition, we evaluated the correlation between KIF23 and clinical features in DLBCL using a chi-square test. As shown in Table 5, different regimens showed different effects on KIF23 expression (*P* = 0.04), while other clinical factors (gender, age, regimen, ECOG, stage, LDH ratio, extranodal sites) were not significantly affecting KIF23 expression.

**Table 5:** Correlation between KIF23 and clinical features in DLBCL.

		KIF23 expression		
	Total number	High	Low	*P*-value
Gender				
male	224	116	108	.2480
female	172	79	93	
Age				
≤50	95	46	49	.7264
>50	325	164	161	
Regimen				
CHOP	180	100	80	**.0426**
R-CHOP	233	106	127	
ECOG				
≤1	296	147	149	.4769
≥2	93	42	51	
Stage				
I-II	188	94	94	.9207
III-IV	218	107	111	
LDH ratio				
low	178	79	99	.1656
high	173	90	83	
Extranodal sites				
=0	238	125	113	.2065
>1	145	66	79	

KIF23 = kinesin family member 23, DLBCL = diffuse large B cell lymphoma.

### 3.9 Molecular mechanism of KIF23 in DLBCL

**Figure 9:**
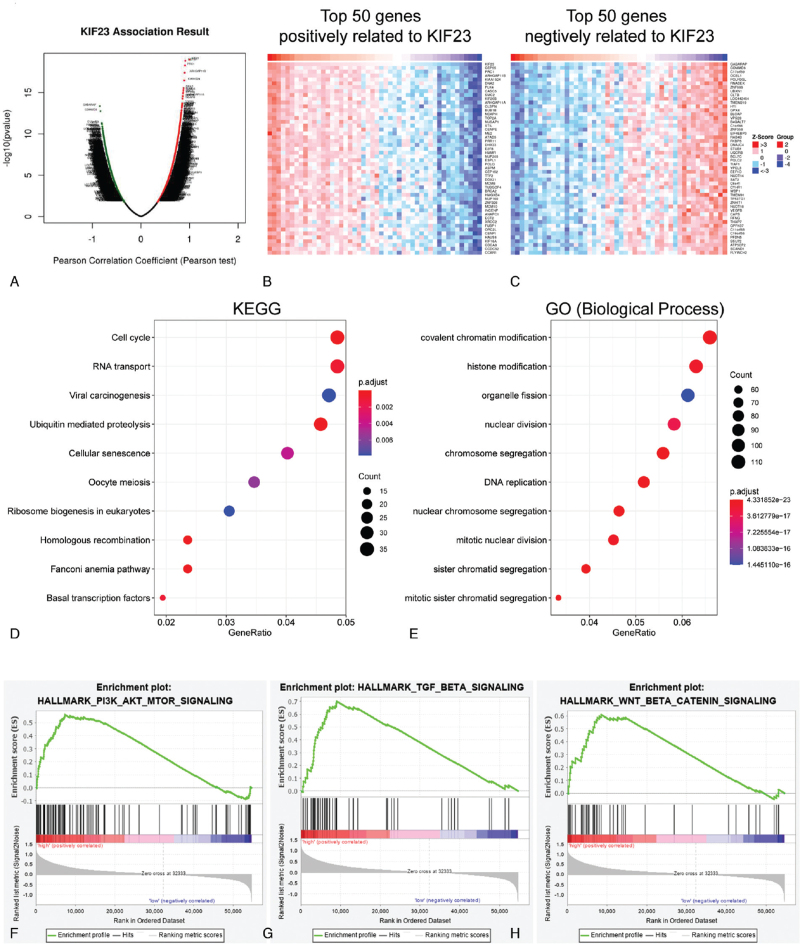
Potential mechanisms of KIF23 in DLBCL. (A) The KIF23 highly correlated genes were identified by Pearson test in DLBCL patients. Heat maps showing the top 50 genes positively (B) and negatively (C) correlated with KIF23 in DLBCL patients. KEGG pathway (D) and GO term (E) in enrichment analysis of genes that were significantly and positively related to KIF23. The relationship between PI3K_AKT_MTOR_SIGNALING (F), TGF_BETA_SIGNALING (G), WNT_BETA_CATENIN_SIGNALING (H) and patients in KIF23 higher group.

KIF23 and its co-expression genes may function together in cells. Hence, we first aim to identify genes that showed significant correlations with KIF23. We used the LinkedOmics database to explore KIF23 co-expression mode in the DLBCL cohort from TCGA. As shown in Fig. 9A, 2875 genes (dark red dots) displayed significantly positive associations with KIF23, while 2671 genes (dark green dots) displayed negative associations (false discovery rate, FDR < .01). Fig. 9B-C showed the top 50 significant genes positively and negatively related to KIF23. Then, we performed KEGG and GO analyses to explore the molecular functions of KIF23 and its co-expression genes in DLBCL. Results showed that KEGG pathways were enriched in cell cycle, RNA transport, viral carcinogenesis, ubiquitin-mediated proteolysis, cellular senescence, oocyte meiosis, ribosome biogenesis in eukaryotes, homologous recombination, fanconi anemia pathway, and basal transcription factors (Fig. 9D). GO annotation displayed that KIF23 may be involved in covalent chromatin modification, histone modification, organelle fission, nuclear division, chromosome segregation, DNA replication, nuclear chromosome segregation, mitotic nuclear division, sister chromatid segregation, and mitotic sister chromatid segregation (Fig. 9E). These results verify that KIF23 play a crucial role in the development of DLBCL.

Most importantly, to explore tumor-related signaling pathways in which KIF23 may participate, we chose the h.all.v7.2.symbols.gmt [Hallmarks] as the gene set database. We divided DLBCL patients from TCGA into two groups according to the median mRNA level of KIF23. Results from GSEA indicated that patients with higher expression of KIF23 showed notably positive correlation with PI3 K/AKT/mTOR_signaling (FDR < .01, Fig. 9F), TGF-beta signaling (FDR < .01, Fig. 9G) and Wnt/beta-catenin signaling (FDR < .01, Fig. 9H). Those three pathways are frequently activated and play important roles in human cancers. These findings suggested that KIF23 may promote DLBCL through PI3 K/AKT/mTOR_signaling, TGF-beta signaling, and Wnt/beta-catenin signaling.

### 3.10 Hypomethylation of the promotor region might be one reason for the upregulation of KIF23 in DLBCL

**Figure 10:**
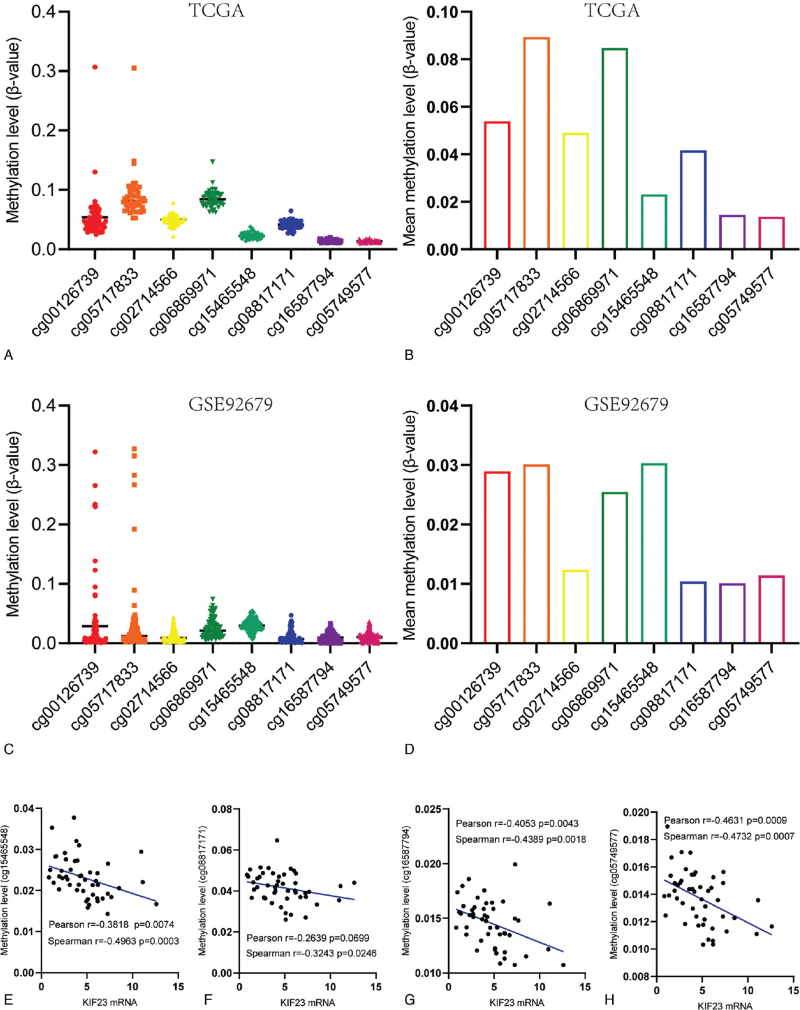
Relationship between KIF23 mRNA expression and its DNA methylation in the promoter region. The methylation levels (A) and the mean methylation levels (B) of 8 CPG probes in the promotor region of KIF23 in the TCGA cohort. The methylation levels (C) and the mean methylation levels (D) of 8 CPG probes in the promotor region of KIF23 in the GSE92679 cohort. The relationship between KIF23 mRNA expression and the CPG probe cg15465548 (E), cg08817171 (F), cg16587794 (G), cg05749577 (H).

It is well known that hypomethylation in the promoter region leads to transcriptional activation. According to the higher expression of KIF23 in DLBCL, we wonder whether hypomethylation is present in the promoter region of KIF23 in DLBCL. However, the methylation levels in the promoter region of KIF23 was not reported in DLBCL. To detect methylation levels in the promoter region of KIF23, we analyzed two DNA methylation profilings (TCGA, n = 48; GSE92676, n = 97). The result from TCGA suggested that CPG probes in the promoter region of KIF23 showed hypomethylation (Fig. 10A). The mean methylation levels of these CPG probes were all less than 0.1 (Fig. 10B). We obtained similar results in the GSE92676 dataset (Fig. 10C-D). To verify the relationship between methylation levels in promoter regions and mRNA expression levels, we combined RNA-seq and DNA methylation profile from TCGA to further analyze. Results confirmed that methylation levels of 4/8 CPG probes (cg15465548, cg08817171, cg16587794, cg05749577) showed significant negative correlations with KIF23 mRNA levels (Fig. 10E-H). Hence, KIF23 hypomethylation in its promoter region might be one reason for its upregulation in DLBCL.

## 4 Discussion

Previous researches indicated that KIF23 was involved in the initiation, development, and progression of tumors. Overexpression of KIF23 was detected in squamous cell carcinoma,^[22]^ gastric cancer,^[23]^ breast cancer,^[24]^ and lung cancer.^[25]^ In this study, integrating four gene expression datasets covering DLBCL patients and normal tissues, we identified that KIF23 expression showed a remarkable discrepancy between normal tissues and tumor tissues of DLBCL. By Kaplan-Meier analysis of four DLBCL profilings (n = 804) with clinical information, we found that the higher expression of KIF23 was consistently associated with poor clinical outcomes. Multivariate analysis indicated that KIF23 might be an independent prognostic factor in DLBCL. Moreover, univariate cox regression analysis revealed that KIF23 higher expression was a prognostic risk factor for patients who received R-CHOP regimen (*P* = .03), in late-stage (*P* = .02), with extranodal sites (*P* = .03) and without extranodal sites (*P* = .04). We also depicted that the higher expression of KIF23 was an adverse factor for decreasing 3, 5, 10-year overall survival. Consequently, we are pragmatic in indicating that KIF23 plays a critical role and leads to shorter lives in DLBCL patients.

PI3 K/AKT/mTOR, TGF-β, and Wnt/beta/catenin signaling pathways are frequently activated in human cancers. These pathways play critical roles in cell proliferation, metabolism, differentiation, invasion/metastasis, and survival.^[26–28]^ In gastric cancer, KIF23 facilitated cell proliferation through directly binding with APC membrane recruitment 1 (Amer) to activate the Wnt/β-catenin signaling pathway.^[29]^ In our study, results of GSEA suggested that DLBCL patients with KIF23 higher expression showed activation of PI3 K/AKT/mTOR, TGF-β, and Wnt/beta/catenin signaling pathways. These findings broaden our knowledge of the molecular mechanism, and we assume that KIF23 may interact with these pathways to initiate and promote DLBCL. Inhibition of PI3 K and mTOR with NVP-BEZ235 can significantly reduce proliferation and the phosphorylation of 4EBP1, therefore inducing cell death^[30]^ in DLBCL. By analyzing gene expression profiles of rituximab (CD20-specific antibody) responsive and unresponsive cell lines of DLBCL, researchers found that rituximab affected not only the expression of genes related to classical pathway but also TGF-β and Wnt signalings.^[31]^ A previous study indicated that FOXP1 could enhance Wnt/β-catenin signaling and improve the sensitivity to Wnt signaling inhibitors in DLBCL.^[32]^ The combination therapy targeting KIF23 and these pathways may provide a new treatment for DLBCL.

DNA methylation is the most common form of epigenetic modification. Dysregulation of DNA methylation is involved in the carcinogenesis of human cancers.^[33–35]^ Previous studies indicated that the hypermethylation of the promoter region was significantly associated with transcriptional silencing.^[36,37]^ Nevertheless, the methylation status of KIF23 in DLBCL is not reported previously. By analyzing the 450 K microarray of DLBCL cohorts, we found that the KIF23 promoter region was hypomethylated. Further study confirmed that methylation levels in the promoter region showed significant inverse correlations with KIF23 mRNA levels in DLBCL. Moreover, according to rigorous screening and validation, we affirm that KIF23 was highly expressed in DLBCL compared to lymphoid tissues. Thus, we supposed that the hypomethylation of the promoter region might be one reason for the higher expression of KIF23 in DLBCL.

However, our study had some limitations. First, although we verified that KIF23 was an adverse prognostic factor in four DLBCL cohorts, the correlations between KIF23 expression and prognosis of patients in different clinical conditions were only analyzed in one GEO dataset (GSE10846 containing 414 samples), since other public datasets lack complete clinical information. A large sample study is required to validate. Second, all the mechanisms of KIF23 identified in DLBCL were based on bioinformatic analysis. Third, due to the lack of DNA methylation profiles of normal lymphoid tissues, our study lacked a comparison of methylation levels in KIF23 promoter regions between normal and DLBCL tissues. What's more, we didn’t confirm relationships between DNA methylation levels in the promoter region of KIF23 and KIF23 mRNA levels in the dataset GSE92676 because GSE92676 lacks the corresponding RNA expression data. Further exploration and validation experimentally in vitro and in vivo were necessary.

## 5 Conclusions

In summary, our results provided evidence of the involvement of KIF23 in DLBCL by unveiling the prognostic values of KIF23 and potentially affected signaling pathways. Further study indicated that the hypomethylation in the promoter region of KIF23 might lead to its upregulation. KIF23 may serve as a potential therapeutic target in DLBCL.

## Acknowledgments

The authors thank Dr. Jianming Zeng (University of Macau) and his team for their kindness of knowledge sharing about bioinformatics.

## Author contributions

**Concept and design:** Yuqi Gong, Jing Zhang, Zhengrong Mao, and Renzhou.

**Conceptualization:** Jing Zhang, Ren Zhou, Yuqi Gong, Zhengrong Mao.

**Data analysis:** Yuqi Gong

**Data curation:** Guoping Ren, Lingna Zhou, Yuqi Gong, Zhe Wang.

**Formal analysis:** Yuqi Gong.

**Funding acquisition:** Jing Zhao, Ren Zhou, Zhengrong Mao.

**Investigation:** Jing Zhao, Lingna Zhou, Yuqi Gong, Zhe Wang.

**Methodology:** Yuqi Gong.

**Resources:** Yuqi Gong.

**Sample collection:** Yuqi Gong, Lingna Zhou, Jing Zhao, Zhe Wang, Guoping Ren.

**Software:** Yuqi Gong.

**Validation:** Yuqi Gong.

**Visualization:** Yuqi Gong.

**Writing – original draft:** Liya Ding, Yuqi Gong.

**Writing – review & editing:** Liya Ding.

**Writing:** Yuqi Gong, Liya Ding.

## Supplementary Material

Supplemental Digital Content

## Supplementary Material

Supplemental Digital Content
