# Supplementary material for: KIF23 is a potential biomarker of diffuse large B cell lymphoma: Analysis based on bioinformatics and immunohistochemistry
Source: Medicine (Baltimore). 2022 Jun 17;101(24):e29312. doi: 10.1097/MD.0000000000029312 (PMC9276187; doi:10.1097/MD.0000000000029312)
Supplement: Supplemental Digital Content [file medi-101-e29312-s001.docx]

**Supplementary file 1**. The detailed information of common DEGs shared by four gene expression profiles

|  | **GSE25638** | | **GSE44337** | | **GSE56315** | | **GSE32018** | |
| --- | --- | --- | --- | --- | --- | --- | --- | --- |
| **Gene** | **log2 FC** | **p value** | **log2 FC** | **p value** | **log2 FC** | **p value** | **log2 FC** | **p value** |
| LUM | 6.50 | 2.19E-17 | 6.21 | 1.47E-05 | 8.20 | 5.74E-50 | 1.15 | 0.005808 |
| GPNMB | 6.58 | 9.06E-16 | 8.02 | 1.04E-07 | 7.92 | 3.54E-58 | 2.04 | 0.000822 |
| CHI3L1 | 5.96 | 7.21E-15 | 5.90 | 0.001772 | 4.10 | 2.02E-19 | 1.04 | 0.011582 |
| VNN1 | 4.43 | 3.09E-14 | 3.13 | 0.026961 | 4.52 | 2.04E-32 | 1.46 | 0.001467 |
| CEP55 | 3.60 | 3.04E-11 | 3.84 | 3.35E-10 | 1.20 | 0.000767 | 1.08 | 0.01775 |
| FNDC3B | 2.56 | 5.27E-11 | 2.27 | 0.006106 | 2.17 | 1.39E-09 | 1.12 | 0.000219 |
| CXCL11 | 4.02 | 7.89E-11 | 3.48 | 0.010231 | 2.42 | 2.87E-12 | 1.51 | 0.011182 |
| CTSD | 2.92 | 2.28E-10 | 3.36 | 0.000139 | 1.97 | 4.41E-22 | 1.24 | 0.006031 |
| PPA1 | 1.29 | 5.15E-10 | 1.33 | 5.63E-05 | 2.05 | 3.75E-35 | 1.01 | 5.93E-05 |
| DTL | 3.46 | 9.52E-10 | 3.85 | 1.18E-06 | 4.01 | 3.92E-24 | 1.62 | 2.98E-06 |
| C17orf58 | 1.31 | 2.84E-09 | 1.74 | 9.13E-05 | 2.09 | 2.40E-17 | 1.46 | 1.11E-07 |
| DEPDC1B | 2.66 | 1.60E-08 | 2.53 | 7.18E-08 | 2.96 | 5.21E-19 | 1.68 | 0.00018 |
| DSCC1 | 2.03 | 1.65E-08 | 2.39 | 3.53E-05 | 1.72 | 4.84E-12 | 1.33 | 0.00091 |
| KIF4A | 2.59 | 2.90E-08 | 3.26 | 1.25E-07 | 1.06 | 0.000493 | 1.12 | 0.000195 |
| TRIP13 | 2.02 | 3.58E-08 | 2.71 | 1.60E-06 | 2.93 | 4.23E-22 | 1.20 | 0.00011 |
| POSTN | 5.14 | 3.79E-08 | 3.90 | 0.015326 | 6.04 | 6.27E-26 | 2.34 | 0.001812 |
| LGALS3 | 3.07 | 4.07E-08 | 4.87 | 1.22E-05 | 5.39 | 1.64E-46 | 1.32 | 0.015028 |
| ECT2 | 2.09 | 4.12E-08 | 3.02 | 5.13E-07 | 2.00 | 1.97E-09 | 1.02 | 7.59E-08 |
| KIF23 | 2.63 | 4.82E-08 | 2.74 | 1.09E-06 | 1.28 | 2.70E-06 | 1.35 | 0.000812 |
| AIMP2 | 1.10 | 4.94E-08 | 1.49 | 4.66E-05 | 1.34 | 1.25E-19 | 1.04 | 1.09E-05 |
| BRCA1 | 1.92 | 6.74E-08 | 2.10 | 3.03E-05 | 2.74 | 6.36E-17 | 1.40 | 0.000226 |
| LDHA | 1.23 | 6.82E-08 | 1.87 | 1.15E-06 | 1.31 | 1.81E-21 | 1.37 | 0.000494 |
| KIF15 | 2.06 | 7.09E-08 | 3.39 | 6.31E-07 | 3.57 | 4.14E-23 | 1.85 | 7.25E-05 |
| MELK | 2.74 | 1.03E-07 | 4.19 | 3.46E-08 | 1.92 | 8.50E-13 | 2.04 | 6.68E-07 |
| TOP2A | 3.83 | 1.07E-07 | 4.89 | 1.59E-11 | 1.98 | 2.26E-07 | 1.98 | 0.007448 |
| MAD2L1 | 2.31 | 1.16E-07 | 3.69 | 9.22E-09 | 1.19 | 9.72E-06 | 1.58 | 0.000205 |
| LRRC59 | 1.02 | 1.31E-07 | 1.14 | 0.000273 | 1.43 | 6.90E-12 | 1.14 | 2.44E-06 |
| TYMS | 3.82 | 1.88E-07 | 5.13 | 2.78E-07 | 1.75 | 1.42E-08 | 2.18 | 1.93E-06 |
| DLGAP5 | 3.32 | 1.98E-07 | 4.11 | 3.56E-10 | 2.30 | 7.25E-09 | 1.36 | 0.000182 |
| NCAPH | 1.80 | 2.31E-07 | 2.59 | 1.57E-08 | 1.45 | 2.26E-05 | 1.18 | 0.001842 |
| FAM83D | 2.14 | 3.07E-07 | 3.18 | 8.28E-07 | 2.68 | 4.79E-16 | 2.17 | 1.53E-07 |
| ANLN | 2.86 | 3.38E-07 | 3.47 | 4.94E-07 | 1.01 | 0.014547 | 1.03 | 0.000819 |
| BUB1 | 2.51 | 3.82E-07 | 3.92 | 3.71E-10 | 1.50 | 6.18E-07 | 1.58 | 0.000456 |
| BYSL | 1.36 | 4.89E-07 | 1.55 | 0.000337 | 1.75 | 4.80E-20 | 1.10 | 6.18E-05 |
| PBK | 3.24 | 5.52E-07 | 4.27 | 6.23E-06 | 2.81 | 8.61E-10 | 1.89 | 7.72E-05 |
| LRR1 | 1.49 | 6.72E-07 | 1.80 | 4.30E-07 | 2.82 | 2.53E-25 | 1.11 | 0.000153 |
| OIP5 | 2.10 | 7.62E-07 | 2.65 | 0.000227 | 1.14 | 2.90E-07 | 1.25 | 0.00344 |
| BCAT1 | 2.28 | 7.96E-07 | 4.50 | 8.21E-06 | 2.77 | 1.39E-17 | 1.59 | 0.000897 |
| KIF14 | 2.53 | 8.07E-07 | 3.34 | 2.80E-06 | 3.10 | 5.37E-20 | 1.37 | 1.91E-05 |
| SPC24 | 1.36 | 8.31E-07 | 1.02 | 0.00029 | 1.15 | 1.61E-05 | 1.14 | 0.000379 |
| KIF2C | 2.36 | 8.60E-07 | 3.81 | 4.51E-07 | 2.27 | 5.24E-15 | 1.53 | 6.30E-06 |
| TTK | 2.58 | 1.03E-06 | 3.03 | 2.77E-06 | 3.92 | 2.51E-23 | 1.80 | 1.94E-06 |
| MRPL15 | 1.29 | 1.03E-06 | 1.27 | 0.000233 | 1.09 | 5.87E-14 | 1.16 | 1.99E-05 |
| CDK1 | 3.40 | 1.22E-06 | 5.42 | 5.84E-10 | 3.35 | 1.64E-13 | 1.58 | 0.000386 |
| IGF2BP3 | 2.99 | 1.27E-06 | 3.27 | 0.000749 | 1.58 | 1.07E-05 | 2.02 | 0.000626 |
| CDC20 | 2.75 | 1.29E-06 | 4.44 | 3.67E-08 | 1.17 | 6.87E-05 | 1.18 | 0.000349 |
| ASF1B | 1.59 | 1.33E-06 | 1.68 | 0.000609 | 1.12 | 3.75E-05 | 1.21 | 9.23E-05 |
| CCNB1 | 2.53 | 1.53E-06 | 4.76 | 9.66E-11 | 1.71 | 7.44E-07 | 1.25 | 0.005318 |
| ZWINT | 2.63 | 1.79E-06 | 5.52 | 2.74E-10 | 1.06 | 0.00067 | 1.67 | 1.63E-05 |
| RAD51AP1 | 1.65 | 1.84E-06 | 2.02 | 7.64E-05 | 2.20 | 2.14E-13 | 1.49 | 0.000247 |
| PCNA | 1.31 | 2.01E-06 | 2.92 | 1.11E-08 | 2.85 | 7.28E-25 | 1.32 | 9.40E-05 |
| CDCA8 | 1.51 | 2.13E-06 | 2.52 | 4.48E-05 | 2.90 | 7.49E-17 | 1.49 | 0.000133 |
| GINS1 | 1.92 | 2.50E-06 | 3.72 | 1.97E-07 | 1.80 | 2.03E-08 | 1.42 | 1.14E-06 |
| NEK2 | 2.27 | 2.50E-06 | 3.58 | 8.98E-08 | 3.23 | 1.29E-16 | 1.54 | 0.000103 |
| ASPM | 3.13 | 2.55E-06 | 4.87 | 6.98E-09 | 2.52 | 2.37E-09 | 1.19 | 0.000945 |
| GMNN | 1.64 | 3.01E-06 | 3.43 | 1.44E-06 | 1.00 | 0.000183 | 1.04 | 0.002535 |
| BUB1B | 2.37 | 3.46E-06 | 4.34 | 7.35E-08 | 1.81 | 2.02E-07 | 1.71 | 6.52E-05 |
| SHCBP1 | 2.58 | 3.95E-06 | 3.42 | 6.54E-05 | 1.64 | 5.30E-05 | 1.37 | 0.002018 |
| CCNE2 | 2.11 | 3.95E-06 | 2.56 | 7.39E-05 | 2.59 | 6.40E-15 | 1.55 | 7.28E-05 |
| HMMR | 2.64 | 4.22E-06 | 4.28 | 3.55E-11 | 3.32 | 8.48E-16 | 1.47 | 0.003425 |
| CDT1 | 1.60 | 4.65E-06 | 1.96 | 6.22E-05 | 2.03 | 1.65E-07 | 1.07 | 0.000999 |
| NUF2 | 2.50 | 4.95E-06 | 3.71 | 2.21E-06 | 1.39 | 0.000163 | 1.62 | 0.000359 |
| TFRC | 1.49 | 5.11E-06 | 1.71 | 0.000396 | 1.87 | 3.65E-20 | 1.73 | 0.000418 |
| RAD51 | 1.54 | 8.50E-06 | 2.21 | 0.000264 | 1.00 | 0.000138 | 1.75 | 6.63E-05 |
| AHCY | 1.07 | 9.24E-06 | 1.37 | 0.003698 | 1.98 | 3.47E-28 | 1.33 | 1.51E-05 |
| SGTB | 1.12 | 1.05E-05 | 1.98 | 6.15E-05 | 2.17 | 6.35E-25 | 1.02 | 0.003452 |
| STIL | 1.43 | 2.04E-05 | 2.18 | 2.31E-06 | 1.54 | 5.16E-11 | 1.39 | 9.11E-07 |
| CENPF | 1.99 | 2.26E-05 | 3.64 | 5.66E-09 | 1.01 | 0.006122 | 1.67 | 1.27E-06 |
| PGD | 1.41 | 3.13E-05 | 2.04 | 3.27E-05 | 2.20 | 6.77E-24 | 1.09 | 0.001326 |
| POLQ | 1.28 | 5.87E-05 | 1.60 | 0.000436 | 2.17 | 1.03E-14 | 1.82 | 2.28E-06 |
| ZNF185 | 1.09 | 0.00012 | 1.97 | 0.006353 | 1.14 | 1.76E-09 | 1.15 | 0.002857 |
| DEPDC1 | 1.88 | 0.000181 | 2.19 | 1.23E-05 | 1.70 | 1.18E-06 | 1.46 | 5.34E-05 |
| ATF3 | 1.30 | 0.000215 | 1.93 | 0.013164 | 2.02 | 2.28E-15 | 1.17 | 0.000428 |
| MYBL2 | 1.36 | 0.00032 | 2.20 | 0.001823 | 2.15 | 2.28E-18 | 1.20 | 0.000376 |
| MCM6 | 1.04 | 0.000394 | 3.44 | 3.39E-06 | 1.74 | 2.45E-13 | 1.28 | 1.40E-05 |
| CDCA7 | 2.31 | 0.00044 | 4.37 | 5.15E-06 | 2.69 | 9.09E-11 | 1.58 | 0.000479 |
| RGS13 | 3.62 | 0.000697 | 5.52 | 0.012773 | 1.76 | 0.000859 | 1.60 | 0.044754 |
| FRK | 1.64 | 0.0008 | 1.36 | 0.048079 | 1.48 | 3.07E-07 | 1.43 | 0.000636 |
| CYB5R2 | 1.76 | 0.001948 | 1.51 | 0.043207 | 1.72 | 2.79E-05 | 1.31 | 0.031207 |
| ADA | 1.13 | 0.011221 | 4.20 | 1.08E-05 | 2.56 | 4.51E-16 | 1.05 | 0.019049 |
| IGHD | -3.30 | 3.71E-11 | -4.14 | 4.60E-05 | -4.09 | 1.77E-24 | -1.62 | 0.0006 |
| RIC3 | -1.02 | 6.70E-09 | -1.25 | 3.42E-06 | -2.37 | 7.90E-38 | -1.58 | 0.000208 |
| JUN | -1.96 | 1.55E-08 | -3.41 | 7.34E-05 | -2.02 | 2.45E-10 | -1.64 | 0.00601 |
| GNG7 | -2.17 | 2.70E-07 | -4.29 | 1.26E-10 | -2.89 | 6.89E-25 | -1.90 | 0.001327 |
| OTUD1 | -1.39 | 1.20E-06 | -2.03 | 0.001807 | -2.19 | 9.53E-24 | -1.05 | 0.000822 |
| CCR6 | -2.89 | 2.67E-06 | -3.16 | 0.004082 | -3.05 | 3.01E-10 | -2.22 | 0.001064 |
| RASGRP2 | -1.69 | 3.54E-06 | -2.99 | 0.001528 | -1.21 | 6.15E-06 | -1.58 | 0.000753 |
| GRAP | -1.06 | 1.51E-05 | -1.09 | 0.005315 | -3.29 | 8.97E-33 | -1.11 | 0.006414 |
| C12orf42 | -1.07 | 2.89E-05 | -2.19 | 6.99E-08 | -1.91 | 9.20E-18 | -1.59 | 0.000137 |
| FCRL1 | -2.28 | 8.73E-05 | -3.79 | 0.001275 | -4.47 | 2.22E-25 | -1.19 | 0.015377 |
| DPEP2 | -1.44 | 9.90E-05 | -3.27 | 5.08E-07 | -2.23 | 3.69E-23 | -1.14 | 0.000133 |
| GAPT | -2.47 | 0.000188 | -4.18 | 0.003035 | -2.00 | 5.19E-06 | -2.15 | 5.87E-05 |
| GLIPR1 | -1.00 | 0.001147 | -2.17 | 0.003464 | -2.35 | 1.52E-20 | -1.37 | 4.39E-06 |
| CYSLTR1 | -1.33 | 0.003325 | -2.16 | 0.015109 | -1.03 | 0.018793 | -1.44 | 0.000266 |
| FCER2 | -1.69 | 0.021004 | -3.26 | 0.002596 | -3.16 | 4.20E-13 | -1.12 | 7.70E-05 |

DEGs: differentially expressed genes
