# Supplementary material for: KIF23 is a potential biomarker of diffuse large B cell lymphoma: Analysis based on bioinformatics and immunohistochemistry
Source: Medicine (Baltimore). 2022 Jun 17;101(24):e29312. doi: 10.1097/MD.0000000000029312 (PMC9276187; doi:10.1097/MD.0000000000029312)
Supplement: Supplemental Digital Content [file medi-101-e29312-s002.docx]

**Supplementary file 2.** The clinical information and the staining index of KIF23 of these 77 DLBCL paraffin-embedded tissues

| Pateint ID | Time(day) | status | KIF23 staining value | KIF23 group |
| --- | --- | --- | --- | --- |
| 201008558 | 1988 | Dead | 12 | high |
| 201028951 | 1865 | Dead | 12 | high |
| 201028980 | 1851 | Dead | 12 | high |
| 201040260 | 4 | Dead | 12 | high |
| 201041046 | 1768 | Alive | 12 | high |
| 201101907 | 1677 | Dead | 12 | high |
| 201147271 | 5 | Dead | 12 | high |
| 201134070/125 | 43 | Dead | 12 | high |
| 201316822 | 336 | Dead | 12 | high |
| 201200186 | 1323 | Dead | 12 | high |
| 201204218 | 1299 | Dead | 12 | high |
| 201219944 | 1214 | Alive | 12 | high |
| 201226326 | 1181 | Dead | 12 | high |
| 201228842 | 1168 | Alive | 12 | high |
| 201234523 | 1094 | Alive | 12 | high |
| 201235753 | 1131 | Dead | 12 | high |
| 201239361 | 1013 | Dead | 12 | high |
| 201239535 | 1110 | Dead | 12 | high |
| 201240112 | 1105 | Dead | 9 | high |
| 201238000 | 1118 | Alive | 9 | high |
| 201244592 | 1084 | Alive | 9 | high |
| 201318589 | 854 | Dead | 9 | high |
| 201326780 | 829 | Dead | 9 | high |
| 201329231 | 791 | Dead | 9 | high |
| 201331075 | 769 | Alive | 9 | high |
| 201335619 | 741 | Alive | 9 | high |
| 201341030 | 880 | Dead | 9 | high |
| 201341768 | 721 | Dead | 9 | high |
| 201344194 | 731 | Dead | 9 | high |
| 201343116 | 735 | Dead | 9 | high |
| 201342024 | 260 | Dead | 9 | high |
| 201343980 | 969 | Dead | 9 | high |
| 201266765 | 41 | Dead | 9 | high |
| 201258513 | 712 | Dead | 9 | high |
| 201346245 | 713 | Dead | 9 | high |
| 201346246 | 676 | Dead | 9 | high |
| 201353003 | 647 | Dead | 8 | high |
| 201359112 | 395 | Dead | 8 | high |
| 201366973 | 397 | Dead | 8 | high |
| 201463139 | 761 | Dead | 8 | high |
| 201474877 | 92 | Dead | 6 | low |
| 201477842 | 1839 | Alive | 6 | low |
| 201032419 | 1630 | Alive | 6 | low |
| 201034106 | 1600 | Alive | 6 | low |
| 201108311 | 6086 | Alive | 6 | low |
| 201113391 | 1335 | Alive | 6 | low |
| 201136153 | 997 | Dead | 6 | low |
| 201158258/346 | 101 | Dead | 6 | low |
| 201412302 | 945 | Alive | 6 | low |
| 201462440 | 945 | Alive | 6 | low |
| 201302673 | 802 | Alive | 6 | low |
| 201303130 | 896 | Alive | 6 | low |
| 201328869 | 889 | Alive | 4 | low |
| 201310226 | 889 | Alive | 4 | low |
| 201311339 | 853 | Alive | 4 | low |
| 201311603 | 840 | Alive | 4 | low |
| 201319179 | 823 | Alive | 4 | low |
| 201320601 | 2252 | Alive | 4 | low |
| 201324734 | 781 | Dead | 4 | low |
| 201327103 | 821 | Alive | 4 | low |
| 201333925 | 748 | Dead | 4 | low |
| 201335690 | 690 | Alive | 4 | low |
| 201339540 | 684 | Alive | 4 | low |
| 201350377 | 5355 | Alive | 4 | low |
| 201351249 | 663 | Dead | 4 | low |
| 201351786 | 647 | Alive | 3 | low |
| 201355779 | 952 | Dead | 3 | low |
| 201358582 | 622 | Alive | 3 | low |
| 201361303 | 616 | Alive | 3 | low |
| 201364174 | 1319 | Alive | 3 | low |
| 201365046 | 1301 | Alive | 2 | low |
| 201202247 | 1258 | Alive | 2 | low |
| 201203432 | 1166 | Alive | 2 | low |
| 201211464 | 1154 | Alive | 2 | low |
| 201229350 | 1110 | Alive | 2 | low |
| 201231087 | 1047 | Alive | 1 | low |
| 201239800 | 1006 | Alive | 1 | low |
